# Geographic Proximity Not a Prerequisite for Invasion: Hawaii Not the Source of California Invasion by Light Brown Apple Moth (*Epiphyas postvittana*)

**DOI:** 10.1371/journal.pone.0016361

**Published:** 2011-01-27

**Authors:** Daniel Rubinoff, Brenden S. Holland, Michael San Jose, Jerry A. Powell

**Affiliations:** 1 Plant and Environment Protection Sciences, University of Hawaii, Honolulu, Hawaii, United States of America; 2 Center for Conservation Research and Training, Pacific Biosciences Research Center, University of Hawaii, Honolulu, Hawaii, United States of America; 3 Essig Museum of Entomology, University of California, Berkeley, California, United States of America; American Museum of Natural History, United States of America

## Abstract

**Background:**

The light brown apple moth (LBAM), *Epiphyas postvittana* (Walker), is native to Australia but invaded England, New Zealand, and Hawaii more than 100 years ago. In temperate climates, LBAM can be a major agricultural pest. In 2006 LBAM was discovered in California, instigating eradication efforts and quarantine against Hawaiian agriculture, the assumption being that Hawaii was the source of the California infestation. Genetic relationships among populations in Hawaii, California, and New Zealand are crucial to understanding LBAM invasion dynamics across the Pacific.

**Methodology/Principal Findings:**

We sequenced mitochondrial DNA (mtDNA) from 1293 LBAM individuals from California (695), Hawaii (448), New Zealand (147), and Australia (3) to examine haplotype diversity and structure among introduced populations, and evaluate the null hypothesis that invasive populations are from a single panmictic source. However, invasive populations in California and New Zealand harbor deep genetic diversity, whereas Hawaii shows low level, shallow diversity.

**Conclusions/Significance:**

LBAM recently has established itself in California, but was in Hawaii and New Zealand for hundreds of generations, yet California and New Zealand show similar levels of genetic diversity relative to Hawaii. Thus, there is no clear relationship between duration of invasion and genetic structure. Demographic statistics suggest rapid expansion occurring in California and past expansions in New Zealand; multiple introductions of diverse, genetically fragmented lineages could contribute to these patterns. Hawaii and California share no haplotypes, therefore, Hawaii is not the source of the California introduction. Paradoxically, Hawaii and California share multiple haplotypes with New Zealand. New Zealand may be the source for the California and Hawaii infestations, but the introductions were independent, and Hawaii was invaded only once. This has significant implications for quarantine, and suggests that probability of invasion is not directly related to geographic distance. Surprisingly, Hawaiian LBAM populations have much lower genetic diversity than California, despite being older.

## Introduction

In recent decades, globalization and the acceleration of anthropogenic trade and travel have increased both the frequency and scope of the introductions of alien species world-wide. Negative impacts of invasive species are evident in agriculture, native ecosystems, and human health [1], [2]. Estimates of the annual financial burden of such invaders on US agriculture and environment are as high as US$120 billion [3].

Invasive phytophagous insects are a major economic concern and constitute a primary target for agricultural quarantine inspections and eradication campaigns [4]. The light brown apple moth (LBAM), *Epiphyas postvittana*, is native to Australia but has been established in England, Hawaii, and New Zealand for 100 years or more [5], [6]. It is a major pest of apple, citrus and grapes in New Zealand and Australia [7] and is known to feed on more than 250 species of plants in many unrelated families, demonstrating a remarkable level of polyphagy. Its possible introduction to the US had therefore been a concern of the USDA (US Department of Agriculture) for many years [8]. Extensive surveys of California moths specifically targeting the family Tortricidae, which includes LBAM, had been underway for decades, and in July 2006 the first LBAM specimen was caught at a black light in Berkeley, northern California. The USDA and CDFA (California Department of Food and Agriculture) initiated pheromone trap surveys in February 2007 and quickly confirmed widespread establishment of LBAM in the San Francisco and Monterey Bay Areas [8], instigating eradication efforts. Although not yet established in California's Central Valley, there is cause for concern that this newly arrived species may become a major pest should it become a resident of this critical agricultural region, or the wine-growing regions of coastal California. Immediately following its discovery on the west coast of the US, an agricultural quarantine was placed on imports to California from Hawaii, where LBAM has been established since at least 1896 [5] yet has never been considered a significant pest.

In Hawaii, LBAM is sparsely distributed, and appears to now be restricted largely to montane wet forest regions. It is unclear why LBAM never became a pest in Hawaii, and is now apparently retreating to a subset of its former range. Perhaps the climate in Hawaii is suboptimal, or parasitoid releases to control other pest moths over the past 100 years have had non-target impacts on LBAM, as they may have had on native Hawaiian moths [9]. Future investigation of this phenomenon of decline in Hawaii may be important in predicting and controlling future impacts of LBAM if it spreads across North America. Justification for quarantine of Hawaiian agricultural products coming into California is based on the assumption that Hawaii served as the source of the LBAM infestation in California, and therefore poses a continued risk. Therefore, the genetic relationships between LBAM in Hawaii, California, and New Zealand are crucial to understanding the invasion dynamics of the moth across the Pacific. Furthermore, elucidation of the invasion pathways for LBAM may be important in understanding the broader factors implicit in the arrival, successful establishment and long-term persistence of agricultural pests since LBAM is a major pest in New Zealand, innocuous in Hawaii, and of as yet undetermined importance in California. However it is certain that in California LBAM is widespread and becoming abundant: intensive pheromone trapping resulted in capture of more than 250,000 specimens from 18 counties across the state in 2007–2009 [8].

In this investigation we specifically address the genetic relationships among invasive LBAM populations in New Zealand, Hawaii and California, what invasion pathways are indicated across these regions, and what patterns of genetic connectivity tell us regarding long-term invaded regions (NZ, HI) compared with recent invasions (CA). Such information is essential in understanding whether there is a relationship between genetic diversity (mtDNA nucleotide and haplotype diversity) and invasion success. For example, in a recent molecular investigation of an explosive gall wasp invasion spanning the Pacific Basin, a single nuclear and mtDNA haplotype was found in multiple populations from Hawaii to China [10]. Further, what does the genetic diversity within and between the regions suggest about the potential for reintroductions to California, should eradication and control efforts prove successful? Finally, what do the genetic relationships suggest about the scientific justification of a costly quarantine on Hawaiian agriculture? In addition, we investigate what predictions can be made regarding the role of genetic diversity in enhancing LBAM's ability to become widespread throughout North America, with or without additional introductions from native or invaded sources. Ultimately, answers to these questions have direct bearing on the interplay between phylogeography, genetic diversity, eradication efforts, quarantine strategy, and public policy.

## Methods

### Sampling

Because Australia represents the natal range of LBAM and likely the greatest diversity of haplotypes, thorough sampling across the continent was outside of the scope of this study and not among the objectives we wished to address regarding the invasive patterns in LBAM. This approach is justified because haplotypes present in invading populations represent subsets of those present in the source populations from the endemic range of the insect, and estimates of mtDNA mutation rates predict that novel haplotypes will not arise in less than two hundred years [11]. We sampled LBAM across its full range on the North and South Islands of New Zealand, and across the Hawaiian Islands. We also sampled extensively over a three-year (2007–2009) period in the recently invaded regions of California (Table S1). Most moths were sampled using pheromone-baited sticky traps that attract males [8]. Some ultraviolet light collection also was used in California (CA), Hawaii (HI) and New Zealand (NZ), but this did not represent the majority of samples in any location. Samples were stored in 90% EtOH in a −20°C freezer until DNA was extracted.

### Laboratory work and data analysis

The head and thorax were dissected and used for genomic DNA extraction, while the remainder of each insect was stored at −80°C as a voucher. Genomic DNA was extracted from all specimens using the DNeasy™ Blood & Tissue kit (Qiagen) following standard protocols. Tissue was digested at 56°C for 24 hours with proteinase K, 200 µl of EB buffer was used to elute the DNA and extracts were stored at −20°C.

Polymerase chain reaction (PCR) was performed using a PTC-200™ (MJ Research, Inc.) We used primers LCO-1490, HCO-2198, Jerry and Pat2 to sequence 1318 base pairs across *COX1* [12], [13] under the following PCR conditions: 2 min at 94°C, 40 cycles of (94°C for 30 s, 50°C for 30 s, and 70°C for 1 min) with a final 70°C extension for 10 min. The PCR products were purified using QIAquick® spin columns (Qiagen) according to the manufacturer's protocol. Cycle sequencing and sequencing was performed at the ASGPB sequencing facility of University of Hawaii at Manoa (http://asgpb.mhpcc.hawaii.edu). For each sample, PCR products were sequenced in both sense and anti-sense strands. All *COX1* sequences were aligned by eye using BioEdit 7.0.9 [14].

TCS 1.21 was used to reconstruct statistical parsimony networks [15]. Pairwise genetic distances were estimated using DnaSP 5.10 [16]. Mismatch plots and Fu's *F*_S_ test statistic were used to evaluate whether demographic expansions or possible secondary contact had occurred for California, Hawaii and New Zealand populations. Mismatch distribution plots comparing pairwise frequency distributions of haplotype distances provide a graphic way to visualize the signature of population expansion. Mismatch plots and associated statistics were produced with Arlequin 3.11 [16], where tau (τ) represents estimated divergence time between populations of unequal size, and theta (θ) is the population parameter of genetic differentiation, and θ = 2Mu, where M is equal to 2N for diploid populations of size N and u is the overall mutation rate at the haplotype level [16].

Maximum likelihood phylogenies were reconstructed using the GTR+Γ model in RAxML (Web-Server version) [17] including only unique haplotypes. DnaSP [18] was used to estimate population-level parameters and their associated variances, including the coefficient of gene differentiation *G*_ST_ [18], [19], pairwise nucleotide diversity π, haplotype diversity *h*, and mean number of nucleotide substitutions per site between populations *D*_xy_ [20]. Traditional population differentiation indices (*F*_ST_) rely on a number of assumptions, including mutation-drift equilibrium and selective neutrality of the markers used. We tested for departures from equilibrium in each population with Tajima's *D* statistic [21], Fu and Li's F* and D* [22], and by plotting the frequency distribution of pairwise differences in mtDNA sequences as proposed by Slatkin and Hudson [23] and Rogers and Harpending [24].

Tajima's *D* contrasts estimates of the population mutation parameter θ, based on π, with those based on the number of segregating sites, for a given sample size. This test statistic is sensitive to demographic effects such as changes in population size. Fu and Li's statistics contrast estimates of θ based on mutations in internal vs. external branches of the gene tree. Designed to assess neutrality, these tests assume that more recent mutations occur near the tips of branches, while older substitutions are internal, and recent mutations confer a selective advantage, and therefore will increase in frequency rapidly under selection. Therefore positive selection results in an excess of identical haplotypes, or mutations in the external branches, and negative values of D* but population size expansions can also result in an excess of external substitutions and negative values of test statistics.

Analysis of molecular variance (AMOVA) was used for hierarchical analysis of the partitioning of *COX1* diversity within and among populations and among regions/groups, using Arlequin 3.11 [16]. We used AMOVA to estimate variance components and *F*-statistic analogues (Φ-statistics), reflecting the correlation of haplotype diversity at different levels of hierarchical subdivision. Significance of Φ-statistics was tested by 1000 permutations of haplotypes among and within populations under the null hypothesis of panmixia. Significance of variance components was also tested using a permutational approach. Nei's average pairwise genetic distances were also computed with Arlequin.

## Results

Altogether 1293 LBAM specimens were included in this population structure analysis, with 147 from across the introduced range on North and South Islands of New Zealand, 448 from Hawaii including all islands on which the species occurs (Oahu, Kauai, Maui, and the island of Hawaii), and 695 covering its recently invaded range in northern California. Three specimens from Australia, where the species is endemic, were included as phylogeographic outgroups. Four additional outgroup taxa were added for the maximum likelihood phylogenetic analysis. We used two genera of moth that, like LBAM, are archipine tortricids, *Syndemis*, (as310 and dr120) sequences were obtained from our work and *Choristoneura* (FSb53 and FSb216) was obtained from GenBank [25] (Table S1).

### Phylogenetic relationships

**Figure 1:**
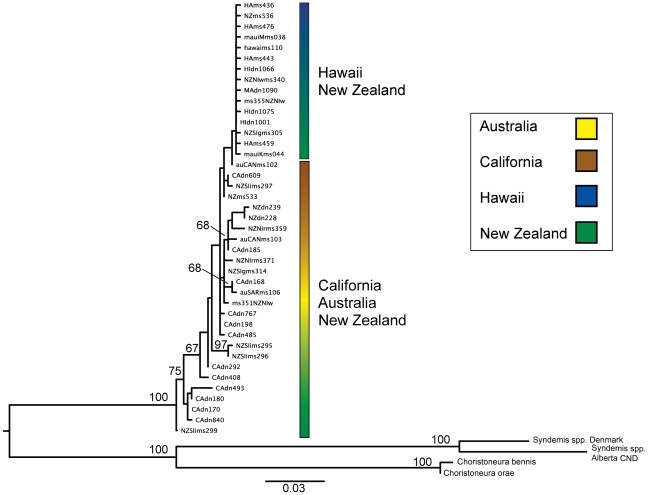
Maximum likelihood reconstruction. Forty-one unique LBAM *COX1* haplotypes were collected in California, Hawaii and New Zealand. RAxML was used for tree reconstruction, with 47 operational taxonomic units, based on 1318 bp of mtDNA *COX1*, with a GTR+Γ substitution model. Colored bars beside clades indicate geographic sources of ingroup samples. Note that overall ingroup topology shows a single main clade containing closely related Hawaii and New Zealand samples, whereas the remainder of the ingroup topology has New Zealand, California and Australian haplotypes mixed throughout.

Maximum likelihood analysis of *COX1* resulted in a single clade with strong bootstrap support confirming the monophyly of all LBAM populations that we tested (Figure 1). The phylogeny reflects the same mixture of Hawaii and New Zealand and California and New Zealand haplotypes as the other analyses.

### Haplotype diversity and population structure

**Figure 2:**
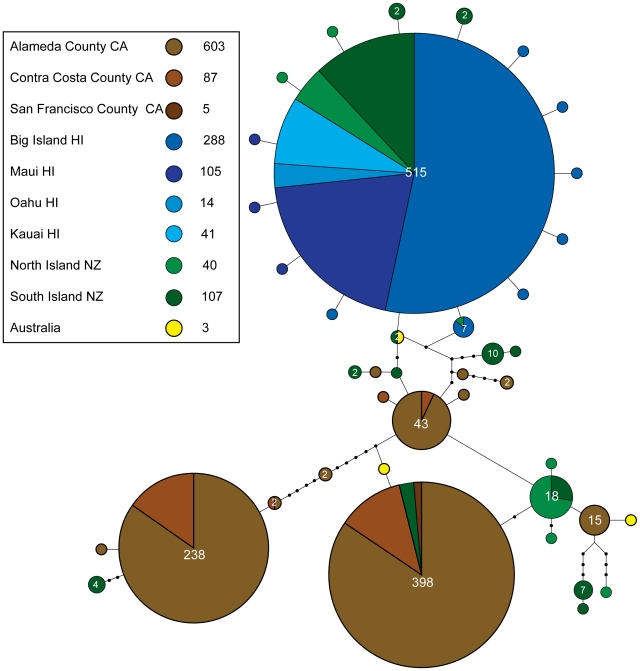
Statistical parsimony network. Network contains 1293 specimens and 1318 bp of *COX1*. Sampling localities include Australia, New Zealand, the Hawaiian Islands and California. Although 12 haplotypes were present in the Hawaiian Island populations, the majority of moths from Hawaii shared a single haplotype, with the brunt of the diversity from these samples coming in the form of singleton samples differing by only a single base change relative to the predominant haplotype, shared by 515 individuals. California populations showed a very different pattern, where although the total number of haplotypes (12) was similar to that of Hawaii (11), the divergence among haplotypes tended to be far deeper, with up to 12 mutational steps, and many specimens shared several different haplotypes. Deeper partitions among haplotypes suggest long population separation times than for the most divergent California and New Zealand haplotypes, whereas all of the moths from Hawaii populations were closely related to one another and to a number of New Zealand haplotypes.

**Figure 3:**
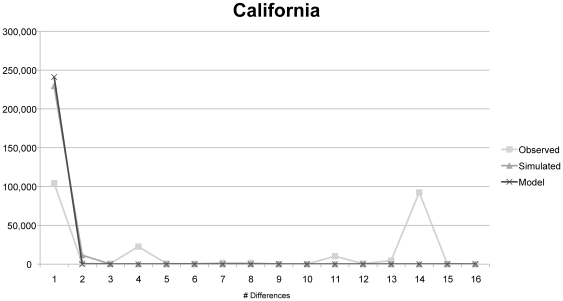
Mismatch distribution plot for California LBAM. Demonstrates strong evidence for demographic expansion (Harpending's raggedness index = 0.4824, τ = 0.000, θ_i_ = 0.000, θ_f_ = 99,999, *p* = 0.000).

**Figure 4:**
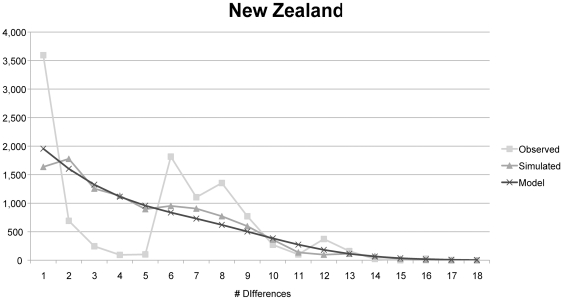
Mismatch distribution plot for Hawaii LBAM. Observed and expected curves were nearly identical (Harpending's raggedness index  = 0.7599, τ = 3.000, θ_i_ = 0.00, θ_f_  = 0.072, *p* = 0.850).

**Figure 5:**
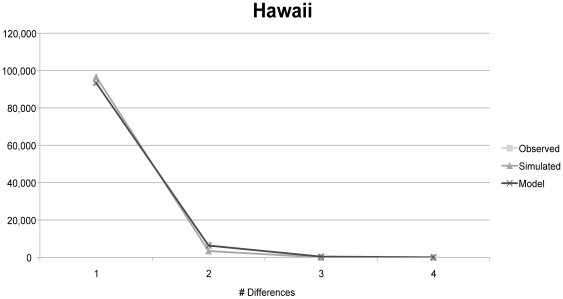
Mismatch distribution plot for New Zealand LBAM. Shows a multimodal distribution of pairwise differences, suggesting past demographic expansion (Harpending's raggedness index  = 0.1121, τ = 7.840, θ_i_ = 0.000, θ_f_  = 4.490, *p* = 0.000).

Test statistics were produced for 1293 *COX1* sequences (GenBank accession numbers HQ534367-HQ535659 and HQ589038-HQ589039) generated for this study, via various approaches including analysis of molecular variance (AMOVA) (Table 1), haplotype diversity (*h*) and nucleotide diversity (π) indices (Table 2), Jukes-Cantor corrected pairwise molecular divergence and *F*_ST_ matrices (Table 3), maximum likelihood tree reconstruction (Figure 1), haplotype networks (Figure 2) and mismatch distribution plots (Figures 3, 4, 5). The genetic diversity and population partitioning patterns for LBAM populations sampled in Hawaii were distinct from patterns observed for the California and New Zealand populations. California and New Zealand *COX1* sequence diversity, as measured by *h* and π, was approximately one order of magnitude higher than for LBAM sampled in Hawaii. California and New Zealand data showed multiple distinct haplotype groups, with relatively deep structure between them, whereas Hawaii had lower haplotype diversity and shallow structure, typically characterized by recent lineage partitioning or ongoing gene flow among islands.

**Table 1:** AMOVA results reveal that molecular variance was approximately equivalent within populations and among populations, and the vast majority of diversity was found among groups plus within populations.

Source of variation	Sum of squares	Variance component	Percent variation	Fixation indices
Among groups	2408.344	1.87712 Va	50.41	*F*_CT_ = 0.49053
Among populations within groups	1380.270	1.82652 Vb	49.05	*F*_SC_ = 0.01051
Within populations	20.296	0.01993 Vc	0.54	*F*_ST_ = 0.49588
Total	3808.910	3.72357		

Significance test was based on 1023 permutations. Va and *F*_CT_: P(random value ≥ observed value)  = 0.00196±0.00136; Vb and *F*_SC_: P(random value ≥ observed value)  = 0.03715±0.00539, populations are the individual Hawaiian Islands, New Zealand's North and South Islands and the California Counties; Vc and *F*_ST_: P(random value ≥ observed value)  = 0.00000±0.00000. Groups are Hawaii, New Zealand, Australia and California.

**Table 2:** Genetic variability of *COX1* sequences (*n* = 1293, 1318 bp) in *Epiphyas postvittana*.

	Australia	California	Hawaii	New Zealand
Sample size	3	695	448	147
No. of haplotypes (N_h_)	3	12	11	17
Haplotype diversity (*h*)	1.00000	0.567	0.066	0.665
Nucleotide diversity (π)	0.00455	0.0046	0.00005	0.0031
No. of segregating sites (*S*)	9	32	10	41
Fu and Li's F*	–	0.486 *p* = 0.10	**−5.731** *p* = 0.02	−1.138 *p* = 0.10
Fu and Li's D*	–	−1.437 *p*>0.1	**−6.349** *p*<0.02	−0.872 *p*>0.1
Tajima's *D*	–	**3.039** *p*<0.01	**−2.0421** *p*<0.05	−1.0552 *p*>0.10
Fu's *F*	–	11.356 *p = 0.96*	**−24.902** *p* = 0.000	−1.707 *p* = 0.35

DNA sequence diversity summary statistics haplotype data were computed using DnaSP. Population demographic statistics shown in bold are statistically significant.

**Table 3:** Pairwise *F*_ST_ values (below diagonal) and genetic distances *D*_XY_ (above diagonal) based on *COX1* sequences (*n* = 1293, 1318 bp).

	Australia	Alameda County	Contra Costa County	San Francisco County	Hawaii	Maui	Kauai	Oahu	South Island New Zealand	North Island New Zealand
Australia	-	0.00497	0.00554	0.00303	0.00380	0.00382	0.00379	0.00379	0.00419	0.00345
Alameda County	0.07943	-	0.00480	0.00360	0.00591	0.00593	0.00591	0.00591	0.00588	0.00522
Contra Costa County	0.12362	0.00569	-	0.00440	0.00621	0.00623	0.00621	0.00621	9,99623	9,99567
San Francisco County	0.42308*	0.15705	0.22619*	-	0.00528	0.00531	0.00531	0.00531	0.00326	0.00286
Hawaii	0.95979*	0.53430*	0.75904*	0.98841*	-	0.00005	0.00003	0.00003	0.00207	0.00171
Maui	0.94610*	0.47918*	0.62443*	0.99219*	0.00375	-	0.00000	0.00000	0.00038	0.00058
Kauai	0.91167*	0.45356*	0.52457*	1.00000*	−0.00700	−0.01047	-	0.00000	0.00038	0.00058
Oahu	0.78010*	0.43537*	0.45639*	1.00000*	−0.03204	−0.03640	0.00000	-	0.00038	0.0058
South Island New Zealand	0.11657	0.30254*	0.33920*	0.47749*	0.28596*	0.18121*	0.12703*	0.08422*	-	0.00010
North Island New Zealand	0.15778	0.28787*	0.33495*	0.57057*	0.65045*	0.48644*	0.34887*	0.24284*	0.02947*	-

Pairwise *F*_ST_ were computed with Arlequin. Pairwise genetic distances were corrected (Jukes–Cantor model) and computed with DnaSP [19]. *F*_ST_ values with asterisk (*) indicate significant *p*-values ≤0.05.

Haplotype diversity levels in California and New Zealand were similar (Table 1), and *F*_ST_ values among their subpopulations were quite high and significant, in the range of 0.29–0.57. Genetic distances and population statistics, including *F*_ST_ values, support isolation of Hawaii and California populations, while simultaneously suggesting that each region has an independent, genetic affinity with LBAM populations from New Zealand (Table 2).

Genetic distance and *F*-statistics demonstrate the greatest divergences in the data are found between California and Hawaii populations, and within New Zealand and within California sampling regions. In contrast, genetic diversity statistics for the California populations suggests they are not completely distinct from New Zealand populations, one haplotype was shared between these regions (10 moths from New Zealand, 388 from California shared a haplotype) as well as between New Zealand and Hawaii populations where two haplotypes are shared (Figure 2). Overall the Hawaii populations were found to be highly similar to one another, and relatively genetically depauperate.

### Haplotype networks

Sampling localities in the TCS network include Australia, New Zealand, the Hawaiian Islands and California (Table S1, Figure 2). Although 11 haplotypes were present in the Hawaiian island populations, the majority of moths collected in Hawaii (432 of 448) shared a single haplotype, with nine unique haplotypes differing from the most common haplotype by only a single base change, and seven individuals shared a haplotype that differed by a single base pair relative to the most common haplotype (Figure 2). The maximum difference between any two Hawaii haplotypes was a single mutational step. California populations showed a very different pattern, although the total number of haplotypes (12) was similar to that found in Hawaii (11), the divergence among haplotypes tended to be far deeper, and dozens to hundreds of moths shared several different haplotypes. Two main haplotypes were shared by 91.5% of moths sampled. But these two dominant haplotypes differed from each other by 12 point mutations. Most of the remaining moths shared a third common haplotype, but again this was relatively divergent compared with the two most common haplotypes. Deeper partitions among haplotypes suggest longer population isolation times than those seen for the Hawaiian data. This may be due to the diversity of the source populations for the California invasion, likely from New Zealand. The difference in genetic diversity may indicate that California was invaded by LBAM multiple times or once with multiple haplotypes. The main Hawaii haplotype was also shared by 67% of the moths from New Zealand. There were also a few moths from New Zealand that shared the most common California haplotype. However, there were no shared haplotypes between California and Hawaii. The absence of shared haplotypes between California and Hawaii is significant in that it strongly suggests that the California introduction did not originate in Hawaii, despite Hawaii's geographic proximity to California relative to New Zealand and California.

### Mismatch distribution plots

Two distinct patterns can be seen in the mismatch distribution analysis, where the California and New Zealand site frequency distributions show significant departures from neutral expectations based on constant population size or a single expansion model, whereas for Hawaii LBAM the observed pairwise site frequency distribution fits the curve based on expected values. Both California and New Zealand show multimodal distributions, the hallmark of demographic expansion and/or multiple invasions. Neutrality test results provide another indication that Hawaii may have undergone a population expansion following a bottleneck.

## Discussion

Because Australia is the native range of LBAM it likely harbors all the haplotypes that occur in the invaded areas (particularly since these invasions occurred in the past 150 years). Thus it is not possible to confirm that New Zealand, rather than Australia, is the source of both the Hawaiian infestation 100 years ago or the more recent California infestations, since Australia must be the ultimate source. While only one and two haplotypes are shared between California and New Zealand and Hawaii and New Zealand, respectively; all the California and Hawaii haplotypes are closely related to New Zealand haplotypes, even if they differ by a few base pairs. In the phylogenetic analysis New Zealand haplotypes fall amongst the California populations and the dominant Hawaiian haplotype. These results suggest that New Zealand may have served as the source for both invasions, and further sampling of New Zealand LBAM may confirm that all invasive haplotypes detected in the United States came from New Zealand. However, the genetic distinctiveness of the Hawaii and California populations is strongly supported by all analyses conducted (Tables 1, 2, 3; Figures 1, 2, 3, 4, 5) and demonstrates that Hawaii could not have been the source of the California infestation. The regions share no haplotypes, but both share haplotypes with New Zealand, and measures of genetic distance between populations consistently indicate that California and Hawaii are the distinct.

One puzzling finding during our sampling across the Hawaiian Islands was the apparent disappearance of LBAM from all lowland habitat and the near extinction of LBAM on the island of Oahu. About ninety years ago (1921), LBAM was recorded down to 600 m (Tantalus) elevation on Oahu (University of Hawaii Insect Museum specimen), on the outskirts of metropolitan Honolulu, and the moth occurred across both volcanoes constituting the Waianae and Koolau mountain ranges. Yet, despite extensive year-round pheromone and light trapping in these same areas and others, we never collected LBAM below 500 m on any island, except for one population on Hawaii Island at 100–450 m and 2 individuals caught on Maui at 136 m and 184 m. Furthermore, the moth has all but vanished from Oahu, no longer occurring in the Koolau Mountains where it was formerly found, and now restricted, ironically, to a single part of the pristine Mt. Kaala Natural Area Reserve for rare endemic species in the Waianae Mountains. This population is of relatively low density compared to both other Hawaiian Islands and California and New Zealand trapping efforts. The dynamics and reasons for the decline of LBAM across the Hawaiian Archipelago are unclear, but trapping revealed only a few localized populations which approached the density of the invasive populations in California and New Zealand, using trap catch as an indicator of population density. This decline bears further examination and may be important in understanding the potential spread and impact that LBAM may, or may not, ultimately have in California.

LBAM haplotype diversity in California was relatively deep, suggesting the presence of at least 12 distinct mtDNA lineages, some of which are quite divergent. Hawaii, despite being an older invasion, has only 11 different haplotypes, and all of them are within one base pair of the dominant haplotype, shared by 96.7% of samples and only one of the other haplotypes is represented by more than one individual. This pattern suggests restricted, shallower genetic diversity than observed in California. Lower genetic diversity revealed in Hawaii could be the result of a founder effect following a population bottleneck associated with the release, in addition to a lack of continued introductions from genetically diverse populations in New Zealand and Australia.

While our sampling from Australia was minimal, all haplotypes must be present in the native range of the moth; so extensive sampling would not conclusively demonstrate the source for California or Hawaii invasions, as long as those same haplotypes, or very similar ones, were also present in the New Zealand samples. This was largely the case, though only one of the haplotypes present in California was represented in our New Zealand samples, California's haplotypes were most similar to those from New Zealand. Based on the relatively high levels of genetic diversity in New Zealand and California, it is likely that there have been multiple invasions from Australia into New Zealand, and at least 12 unique female haplotypes have invaded California.

The data support the idea that Hawaii was infested from Australia or New Zealand over 100 years ago, and a separate and recent introduction or series of introductions from New Zealand or Australia to California took place with no contribution from Hawaii. This result is surprising since Hawaii and California are not only geographically much more proximate but, as part of the same country, they share a brisk trade in goods and air traffic; California, in addition to being the closest continental landmass to Hawaii, is by far Hawaii's leading trading partner for agricultural goods and tourism. Thus, the data suggest that invasion pathways are not necessarily governed by levels of trade or geographic distance. Rather the level of the infestation in the source region appears to play the most significant role in determining invasion risk. For the past 100 years LBAM has been established in Hawaii and has still not made it to California. Yet, at least 12 LBAM haplotypes have successfully colonized California from a New Zealand/Australian source.

### Management implications

The number of haplotypes present in California suggests that there have either been multiple invasions in a short period of time, or a single large infestation (e.g. an infested shipment of produce or horticultural products with hundreds of eggs or small larvae). The California invasion has maintained high genetic diversity, and this could be critical to the long-term persistence of LBAM in this novel habitat. Typically the homogenizing impacts of a strong founder event result in lower genetic diversity, LBAM's Californian diversity may help the moth adjust to adverse conditions. Regardless of whether the California infestation is eliminated, greater attention should be focused on inspections of imports, since other pests may arrive in a similar manner from New Zealand and Australia and be equally inconspicuous. Based on LBAM's apparently innocuous presence in Hawaii, there is a possibility that the moth may not cause severe damage in California, and after a period of population increase it may suffer the same decline that is typical of many invasive species over time [26].

### Concluding remarks, implications and future work

From a scientific perspective, arguments for agricultural quarantine against Hawaii for LBAM appear unsubstantiated. Despite 100 years of intense trade, there is no evidence that LBAM from Hawaii has become established in California. Thus, California's quarantine of Hawaiian exports should be reconsidered. Despite the fact that Hawaii is geographically much closer to California, and is part of the same country, New Zealand or Australia is the source of the California invasion. This result demonstrates that trade trumps geographic and political boundaries in facilitating the spread of invasive species. Future efforts to control invasions through these pathways are essential, not just for LBAM but also for other pests from New Zealand and Australia that pose a risk to California's environmental and agricultural biosecurity. The data suggest that Hawaii was invaded only once, or possibly multiple times from a single geographic, genetically homogeneous population. Furthermore the data suggest that beyond distances that species can traverse naturally, whether actively or passively, geographic proximity does not enhance likelihood of invasion; New Zealand or Australia is the likely source of the California introduction, the distance from New Zealand is 11,073 km (6,882 miles) and from Australia is 13,203 km (8,204 miles) whereas Hawaii is 4,034 km (2,506 miles) from California. Interestingly, survey data anecdotally support a recent population crash of LBAM in the Hawaiian Islands, and this may be a factor in the observed depauperate genetic patterns.

Population genetic theory predicts that older established populations may have more pronounced genetic structure, due to opportunities for barriers to gene flow to arise, yet this pattern was not observed. LBAM sampling should be continued in California to establish a genetic baseline, and to try to detect whether introduction is ongoing. The molecular dataset presented could be used in a comparative framework for future sampling, from both additional invaded and native sources. In addition it would be worthwhile to obtain and compare samples from across the native range of LBAM in order to begin to understand the phylogeographic sources of invasive haplotypes characterized herein, in an effort that promises ultimately to shed light on the mechanisms of transport and release of this global pest species.

## Supporting Information

Table S1 **Voucher identities, sample locations, collectors, and GenBank Accession numbers for LBAM (** ***Epiphyas postvittana*** **) specimens analyzed in this study.**
(DOCX)
