# Supplementary material for: Geographic Proximity Not a Prerequisite for Invasion: Hawaii Not the Source of California Invasion by Light Brown Apple Moth (Epiphyas postvittana)
Source: PLoS One. 2011 Jan 27;6(1):e16361. doi: 10.1371/journal.pone.0016361 (PMC3029344; doi:10.1371/journal.pone.0016361)
Supplement: Table S1 — Voucher identities, sample locations, collectors, and GenBank Accession numbers for LBAM ( Epiphyas postvittana ) specimens analyzed in this study. (DOCX) [file pone.0016361.s001.docx]

Table S1

| **Voucher IDs** | **Locality** | **Date** | **Collector** | **GenBank accession numbers** |
| --- | --- | --- | --- | --- |
| ms102-103; 106 | Australia: Canberra | April 2008 | Hobern | HQ534367-HQ534369 |
| ms076-078 | California: Alameda Co., Berkeley | July 2007 | Powell | HQ535028-HQ535030 |
| dn288-722 | California: Alameda Co., Berkeley | July 2009 | Powell | HQ534448-HQ534835 |
| dn904-933 | California: Alameda Co., Berkeley | July 2009 | Rubinoff | HQ535000-HQ535027 |
| ms097-099; ms147-153 | California: Alameda Co., Berkeley | July 2008 | Rubinoff | HQ535036-HQ535038; HQ535041-HQ535046 |
| dn859-892 | California: Alameda Co., Berkeley | July 2009 | Rubinoff | HQ534966-HQ534999 |
| dn771-858 | California: Alameda Co., Berkeley | May/June 2009 | Powell | HQ534881-HQ534965 |
| dn168-191; dn252-283 | California: Alameda Co., Berkeley | May 2009 | Powell | HQ534370-HQ534389; HQ534420-HQ534447 |
| ms170-177 | California: Alameda Co., Berkeley | Sept/Oct 2008 | Powell | HQ535047-HQ535054 |
| ms100-101 | California: Contra Costa Co., Albany | April 2008 | Oboyski | HQ535039-HQ535040 |
| ms223-232 | California: Contra Costa Co., Albany Hill | April 2008 | Rubinoff | HQ535055-HQ535064 |
| dn723-770 | California: Contra Costa Co., Richmond | Aug 2009 | Ghajar | HQ534836-HQ534880 |
| dn192-226 | California: Contra Costa Co., Richmond | June 2009 | Ghajar | HQ534390-HQ534419 |
| ms079-083 | California: San Francisco Co., San Francisco | July 2007 | Powell | HQ535031-HQ535035 |
| ms416-474 | Hawaii: Island of Hawaii, Kona Kaloko Drive | June 2009 | San Jose | HQ535289-HQ535331 |
| dn934-1063 | Hawaii: Island of Hawaii, Saddle Road | Aug 2009 | Rubinoff Eiben San Jose | HQ535065-HQ535119 |
| ms094-95; ms117-135; | Hawaii: Island of Hawaii, South Kona | Feb/Mar 2008 | Follet | HQ535224-HQ535225;  HQ535237-HQ535255 |
| ms476-ms498 | Hawaii: Island of Hawaii, South Kona Milolii Road | June 2009 | San Jose | HQ535332-HQ535352 |
| ms104 | Hawaii: Island of Hawaii, Volcano Colony |  |  | HQ535226 |
| ms067-68 | Hawaii: Island of Hawaii, Volcano Village | Aug 2009 | LeBlanc | HQ535212-HQ535213 |
| ms234-252 | Hawaii: Island of Hawaii, Volcano Village | Oct 2008 | Rubinoff | HQ535270-HQ535288 |
| ms154-167 | Hawaii: Island of Hawaii, Volcanoes National Park | Oct 2008 | Rubinoff | HQ535256-HQ535269 |
| ms084-093;  ms107-116; dn1064-1089 | Hawaii: Island of Hawaii Waimea | Feb/Mar 2008 | Follet | HQ535214-HQ535223; HQ535227-HQ535236; HQ535120-HQ535145 |
| ms061-065 | Hawaii: Kauai, Alakai Trail Head | Aug 2007 | Rubinoff | HQ535461-HQ535465 |
| ms258-269; dn1153-1169 | Hawaii: Kauai, Kalalau Lookout | Feb 2009 | Rubinoff Schmitz San Jose | HQ535472-HQ535483; HQ535458-HQ535460 |
| ms070-075 | Hawaii: Kauai, Kokee | Aug 2007 | Rubinoff | HQ535466-HQ535471 |
| ms279-294 | Hawaii: Kauai, Waimea Canyon | Feb/Mar 2009 | Rubinoff Schmitz San Jose | HQ535484-HQ535498 |
| ms381 | Hawaii: Maui, Kokomo | Mar/Apr 2009 | Haines | HQ535456 |
| ms383 | Hawaii: Maui, Hana Hwy marker 2 | Mar/Apr 2009 | Haines | HQ535457 |
| dn1090-1150 | Hawaii: Maui, Kula | Oct 2008 | Fukuda | HQ535353-HQ535396 |
| ms041-060; ms136-146 | Hawaii: Maui, Kula Agriculture Station | Aug 2007 | King | HQ535426-HQ535443; HQ535444-HQ535454 |
| ms011-040 | Hawaii: Maui, Makawao Forest Reserve | Aug 2007 | King | HQ535397-HQ535425 |
| ms257 | Hawaii: Maui Waihee Trail | Jan 2008 | Haines | HQ535455 |
| ms211-ms220 | Hawaii: Oahu, Pahole NAR | Jan 2009 | Joe | HQ535503-HQ535512 |
| dn1174-1179 | Hawaii: Oahu, Pahole NAR | Nov 2009 | Joe | HQ535499-HQ535502 |
| ms511-522 | New Zealand: South Island, Greymouth | March 2009 | Rubinoff | HQ535628-HQ535638 |
| ms355-375 | New Zealand: North Island, Roto Rua | March 2009 | Rubinoff | HQ535590-HQ535610 |
| ms336-354 | New Zealand: North Island, Waitomo | March 2009 | Rubinoff | HQ535571-HQ535589 |
| ms305-315 | New Zealand: South Island, Greymouth | March 2009 | Rubinoff | HQ535540-HQ535550 |
| ms376-377 | New Zealand: South Island, Queenstown | April 2009 | Rubinoff | HQ535611-HQ535612 |
| ms378-380 | New Zealand: South Island, Invercargill | March 2009 | Rubinoff | HQ535613-HQ535615 |
| dn228-251; ms326- 335; ms499-510 | New Zealand: South Island, Okura | March 2009 | Rubinoff | HQ535513-HQ535529; HQ535561-HQ535570; HQ535616-HQ535627 |
| ms316-325; ms535-546 | New Zealand: South Island, Portobello Otago Peninsula | March 2009 | Rubinoff | HQ535551-HQ535560; HQ535649-HQ535659 |
| ms295-304; ms523-534 | New Zealand: South Island, Te Anau | March 2009 | Rubinoff | HQ535530-HQ535539; HQ535639-HQ535648 |
| FSb53 | *Choristoneura biennis* |  |  | DQ79287 |
| FSb216 | *Choristoneura orae* |  |  | DQ79286 |
| as310 | *Syndemis* sp. Denmark | May 2007 | Karsholt | HQ589039 |
| dr120 | *Syndemis* sp. Sherwood Park, Alberta CND | May 2001 | Pohl | HQ589038 |
